# Overexpression of CD59 inhibits apoptosis of T-acute lymphoblastic leukemia via AKT/Notch1 signaling pathway

**DOI:** 10.1186/s12935-018-0714-9

**Published:** 2019-01-08

**Authors:** Yanfei Jia, Yan Qi, Yunshan Wang, Xiaoli Ma, Yihui Xu, Jun Wang, Xiaoqian Zhang, Meihua Gao, Beibei Cong, Shuyi Han

**Affiliations:** 1 Medical Research and Laboratory Diagnostic Center, Jinan Central Hospital Affiliated to Shandong University, 115 Jie Fang Road, Jinan, Shandong 250013 People’s Republic of China; 2 Department of Clinical Laboratory, Qingdao Municipal Hospital, Qingdao, Shandong People’s Republic of China

**Keywords:** CD59, T-acute lymphoblastic leukemia, Apoptosis, AKT, Notch1

## Abstract

**Background:**

T-acute lymphoblastic leukemia (T-ALL) was a hematological malignancy characterized by the accumulation of immature T cells in bone marrow and peripheral blood. In this study, we tried to explore the physiological role of CD59 in T-ALL.

**Methods:**

In this study, we collected the bone marrow samples from 17 T-ALL patients and 38 healthy participants to find differences in CD59 expression patterns. Then, CD59 was over-expressed in T-ALL cell line Jurkat, and its biological functions were detected. In addition, in order to understand the active site of CD59, the Trp40 was mutated. Further, we constructed a mouse model by transplanting Jurkat cells into the nude mice to verify the function of CD59 in vitro. At last, mechanism studies were performed by western blot.

**Results:**

We found that the proportion of T lymphocytes expressing CD59 in bone marrow of T-ALL patients was significantly higher than that of healthy individuals. Then, we found that the overexpression of CD59 in Jurkat cells was beneficial to the cell survival by inhibiting apoptosis and promoting IL-2 secretion. In this process, Trp40 of CD59 was a key functional site. Further, the high expression of CD59 inhibited apoptosis of bone marrow and peripheral blood cells, and promoted IL-2 secretion in mouse model. At last, mechanism studies showed that the activation of AKT, STAT5 and Notch1 signaling pathways in Jurkat cells, may be involved in the regulation of apoptosis by CD59; and mutation in the Trp40 affect the interaction of CD59 with these signaling pathways.

**Conclusions:**

In conclusion, CD59 inhibited apoptosis of T-ALL by regulating AKT/Notch1 signaling pathway, providing a new perspective for the treatment of T-ALL.

**Electronic supplementary material:**

The online version of this article (10.1186/s12935-018-0714-9) contains supplementary material, which is available to authorized users.

## Background

T-acute lymphoblastic leukemia (T-ALL) was a hematological malignancy characterized by the accumulation of immature T cells in bone marrow and peripheral blood, of which clinical manifestations might include extramedullary leukocytosis of lymph nodes and other organs, frequently central nervous system infiltration, and mediastinal masses from thymus [1, 2]. T-ALL accounted for 15% of childhood acute lymphoblastic leukemia and 25% of adult acute lymphoblastic leukemia [3, 4]. Even with aggressive treatment regimens such as high-dose multi-drug chemotherapy or hematopoietic stem cell transplantation which caused enormous acute and long-term side effects, about 15% of children and 40% of adult patients still relapse, owing to treatment resistance, and their survival prospects were dim [5]. Targeted therapy of monoclonal antibodies against lymphocyte surface antigens played an important role in the treatment of leukemia and other tumor types [6].

CD59 was a small (18–20 kD), highly glycosylated and glycosylphosphatidylinositol (GPI) anchoring protein that was widely distributed on various cell surfaces, including hematopoietic producing cells, non-hematopoietic producing cells, tissue cells, etc. [7]. CD59 was first recognized as a complement regulatory protein that blocked the binding of C9 or C8 to the C5b complex, and inhibited the formation of membrane attack complex (MAC) on the cell surface [7]. The tumor cells used this characteristic to evade immunity. One research had shown that up-regulation of CD59 in cancer stem cells (CSCs) allowed it avoid complement attack, and silence of CD59 can completely eliminate tumors in a mouse model in which CSCs were implanted [8]. Subsequent studies detected that most solid tumors expressed CD59 at a markedly high level compared to adjacent normal tissues, of which stage and prognosis were related to the CD59 levels, including breast cancer, non-small cell lung cancer, colon cancer, etc. [9–11]. In this study, we sought to find out the role of CD59 in the progression of T-ALL, which was rarely studied.

During its cell surface expression, the amino-terminal 25 amino acids and the carboxy-terminal 26 amino acids of human CD59 precursor which was a single peptide consisting of 128 amino acids were truncated, then mature form of CD59 consisted 77 amino acids, starting with Leu, terminated with Asp, folded through five intrachain disulfide bonds, and glycosylated at Asn18 and Asn77 to joint GPI [12–17]. The tertiary structure of CD59 was known, but it was still confusing for the active site relative to MAC [17]. In 1995, Nakano et al. [18] used the synthetic peptide method to consider amino acid residues 27–38 as the active site for CD59 binding to C8 or C9. In 1997, Bodian et al. [19] perceived that Phe23, Cys39, Trp40 and Leu54 formed a hydrophobic trench, and constituted an active region. Trp40 was located on the bottom of the hydrophobic trench [19]. Subsequently, Zhu’s team confirmed that Trp40 was an important functional residue for complement function of CD59, and CD59 molecule enhanced its complement inhibitory function after the adjacent 39 and 41 sites were mutated to Trp [20]. In this study, we still used point mutation technique to further explore the functional impact to Trp40 mutation on CD59.

## Methods

### Flow cytometry analysis of bone marrow samples in patients with T-ALL

Bone marrow samples of 17 T-ALL patients and 38 healthy participants were collected from the Department of Hematology, Jinan Central Hospital from January 2012 to June 2016. None of the participants had other tumors or serious diseases. All T-ALL patients were clinically diagnosed at this hospital, and did not receive any chemotherapy, radiotherapy or other treatment prior to bone marrow collection. All clinical and pathological information (including gender, age and disease stages) were from the cases. All participants were informed of the purpose and needs of this study, and provided informed consent.

The bone marrow samples were vortexed for 3 s or manually reversed 10 times. And 50 μl of bone marrow sample was added to the bottom of the flow tube by reverse sampling method, prior to this, 20 μl each of Anti-Human CD59-FITC (1935069, Invitrogen, USA), Anti-Human CD3-PE (1937981, Invitrogen, USA) and Anti-Human CD45 PerCP-Cyanine5.5 (4277917, eBioscience, USA) had been added to the bottom. Vortex for 3 s and incubate at room temperature for 15 min in the dark. 2 ml of 1 × hemolysin was added to each tube. Vortex for 3 s, and incubate at room temperature for 15 min in the dark again. Then, centrifuge 300×*g* for 5 min, discard the supernatant and wash once with 2 ml of PBS per tube. Add 500 μl of PBS to each tube, mix by shaking, and immediately check on using machine. Statistical analysis was performed using Flowjo software.

### Wild or mutant CD59 expressed Jurkat cells

The Trp40 (W40) and Lys41 (K41) sites were selected for point mutations (Table 1). After obtaining and purifying the gene of interest, it was digested and ligated into a lentiviral vector which infected to Jurkat cells to obtain Jurkat cells stably expressing the wild or mutant human CD59. The specific procedures were as follows: Human CD59 cDNA-pALTER recombinant plasmid containing T7, T3 RNA polymerase promoter, *Eco*RI restriction site, signal peptide and CD59 cDNA sequence was provided by the Harvard Medical School of the United States. Using this plasmid as a template, overlap extension PCR was performed to obtain the desired gene sequence. The primers were shown in Table 2. pT7 and T3 were normal primers, M1F and M1R were the W40 site mutation primers (M1), M2F and M2R were K41 site mutation primers (M2). pT7 and M1R (or M2R) were used as primers for PCR1: predenaturation at 94 °C for 3 min; 25 cycles of denaturation at 94 °C for 30 s, annealing at 42 °C for 45 s, and elongation at 72 °C for 45 s; finally elongation at 72 °C for 7 min. M1F (or M2F) and T3 were used as primers for PCR2: the amplification conditions were the same as those of PCR1. The template for PCR3 was an equimolar mixture of PCR1 and PCR2 which were purified by a DNA Gel Extraction Kit (Bio-Dev, China). pT7 and T3 were used as primers for PCR3: except that the annealing temperature was 46 °C, other amplification conditions were the same as those of PCR1. The PCR3 product was separated by 1% agarose gel electrophoresis and purified using a DNA Gel Extraction Kit, then was M1 human CD59 (or M2 human CD59) sequence. Using the plasmid as a template, and pT7 and T3 as primers, the amplification conditions were the same as those of PCR3, and normal human CD59 sequences were obtained.

**Table 1:** Wild and mutant human CD59

Protein	37	38	39	40	41	42	43	44	45	46
Wild	N	K	C	W	K	F	E	H	C	N
M1	N	K	C		K	F	E	H	C	N
M2	N	K	C	W		F	E	H	C	N

**Table 2:** Primers for CD59 gene

Primer	Sequence (5′–3′)
pT7	TAA TAC GAC TCA CTA TAG GC
T3	ATT AAC CCT CAC TAA AGG GA
M1F	A GTG TAT AAC AAG TGT AAG TTT GAG CA
M1R	TG CTC A AA CTT A CA CTT GTT ATA CAC T
M2F	GTG TAT AAC AAG TGT TGG TTT GAG CAT TGC
M2R	ATG CTC AAA CTT CCA CTT GTT ATA CAC TTG

Three gene sequences were ligated to the lentiviral vector pCDH-CMV-MCS-EF1-GFP+Puro (VT8070, YouBio, China), respectively. Subsequently, virus particles were collected according to the manufacturer’s instructions for the Lenti-v pak Packaging Kit Protocol (TR30037, Origene, USA). Finally, the lentivirus were infected into Jurkat cells. The expression efficiency of the target gene in the cells was examined using fluorescence microscope, flow cytometry analysis, real-time quantitative PCR and western blot.

### Cell counting kit-8 (CCK8) assay

CCK8 was used to assess cell proliferation. The cell density in the logarithmic growth phase was adjusted to 5 × 10^4^ cells/ml, and 100 μl was plated in the wells of 96-well plates. A total of five parallel replicates were set for each group. Incubate at 37 °C in a humidified atmosphere with 5% CO_2_ for 24 h, 48 h, 72 h. At a specific time point, the culture medium was removed and 100 μl of fresh medium containing 10 μl CCK8 solution was added to each well, followed by incubation at 37 °C for 2 h. The absorbance (OD value) of each well at 450 nm was measured with a microplate reader.

### Blue trypan exclusion assay

5 × 10^3^ cells/100 μl in the logarithmic growth phase were plated in the wells of 96-well plates and cultured at 37 °C in a humidified atmosphere with 5% CO_2_. A total of 3 parallel replicates were set for each group. After 48 h of culture, all cells in each well were collected by centrifugation at 300×*g* for 5 min. Further remove the supernatant, resuspend the cells in appropriate amount of Hanks solution, and adjust the cell density to 1 × 10^5^/ml. One drop of freshly prepared trypan blue dye was added to each 0.1 ml cell suspension, and stained for 3–5 min at room temperature. Take a drop of the stained cell suspension, and observe under high magnification. The dead cells were pale blue, enlarged and dull. Live cells were not colored, maintaining their normal morphology and shine.

### Dye release assay

Dye release assay determined the sensitivity of cells to complement-mediated cytolysis. The higher the rate of dye release, the more sensitive the cells were. Human fresh serum was used as a source of complement. The results were expressed as an average of three experiments.

$${\text{Dye}}\;{\text{release}}\;{\text{rate}}\, = \,{\text{supernatant}}\;{\text{fluorescence}}\;{\text{intensity}}/\left( {{\text{Supernatant}}\;{\text{fluorescence}}\;{\text{intensity}}\, + \,{\text{lysate}}\;{\text{fluorescence}}\;{\text{intensity}}} \right)\, \times \,100\% .$$

2 × 10^4^ cells/100 μl in the logarithmic growth phase were plated in the wells of 96-well plates. After 24 h of incubation, 22 μl of NaBH4 stock solution (200 mM) was added to each well, and incubation was continued for 20 min. Add 100 μl of BCECF application solution (2 μg/ml) to each well, and incubate in a 37 °C shaker for 30 min. All cell mixture was centrifuged at 300×*g* for 5 min at room temperature, and cells precipitations were washed 2 times with standard buffer. Add 200 μl of RPMI 1640 medium containing 5% fresh human serum, and incubate at 37 °C for 30 min. All cell culture fluides were centrifuged, and the supernatant was added to 2 ml of PBS. Fluorescence intensity was measured at 503 nm for excitation and 530 nm for emission. Add 50 μl of cell lysate to the cell precipitations, mix by pipetting, lyse at 4 °C for 30 min. The cell lysate was added to 2 ml of PBS, and its fluorescence intensity was measured under the same conditions.

### Apoptosis detection

After 48 h of culture, cells were harvested and washed twice with cold PBS. Resuspend the cells with 1× binding buffer, and adjust the cell density to 10^6^/ml. 5 μl of fluorochrome-conjugated Annexin V was added to 100 μl of cell suspension, and incubated in the dark for 10 min at room temperature. Then add 5 μl of PI and 400 μl of PBS, gently mix, and immediately place on a flow cytometer for detection. Flowjo software was used to all data analysis.

### Western blot

After 48 h of culture, cells were collected and lysed by RIPA lysate at 4 °C for 20 min. After centrifugation of the lysate at 12,000×*g* for 10 min, the supernatant was boiled for 5 min, ice-bathed for 3 min, and separated by a 12% SDS-PAGE gel and then transferred to a PVDF membrane. The PVDF membrane was blocked with 5% skim milk for 1 h at room temperature, and incubated overnight with primary antibody at 4 °C, followed by incubation of the secondary antibody at room temperature. Wash twice with TBST before the ECL reagent was used to perform chemiluminescence. QUANTITY ONE software was used to quantify chemiluminescence.

The primary antibodies used in this study were as follow: anti-BCL2 (1:2000, Mouse polyclonal antibody, 12789-1-AP, Proteintech, Manchester, UK); anti-Bax (1:1000, Rabbit polyclonal antibody, 23931-1-AP, Proteintech, Manchester, UK); anti-actin (1:1000, Rabbit polyclonal antibody, AF7018, Affinity BioReagents, Golden, CO, USA); anti-CD59 (1:1000, Rabbit polyclonal antibody, #65055, Cell Signaling Technology, Beverly, MA); anti-Caspase 3 (1:1000, Rabbit polyclonal antibody, 25546-1-AP, Proteintech, Manchester, UK); anti-Bim (1:1000, Rabbit polyclonal antibody, DF6093, Affinity BioReagents, Golden, CO, USA); anti-p-ERK (1:500, Rabbit monoclonal antibody, Thr202/Tyr204, #4370, Cell Signaling Technology, Beverly, MA); anti-ERK (1:500, Rabbit monoclonal antibody, #9102, Cell Signaling Technology, Beverly, MA); anti-p-AKT (1:1000, Mouse monoclonal antibody, Ser473, 66444-1-Ig, Proteintech, Manchester, UK); anti-AKT (1:500, Mouse monoclonal antibody, #9272, Cell Signaling Technology, Beverly, MA); anti-p-STAT5 (1:500, Rabbit monoclonal antibody, Tyr694, #9314, Cell Signaling Technology, Beverly, MA); anti-STAT5 (1:500, Rabbit monoclonal antibody, #25656, Cell Signaling Technology, Beverly, MA); anti-HEY1 (1:1000, Rabbit polyclonal antibody, DF12079, Affinity BioReagents, Golden, CO, USA); anti-Notch1 (1:1000, Rabbit polyclonal antibody, AF5037, Affinity BioReagents, Golden, CO, USA).

### Quantitative real-time PCR (qRT-PCR)

Total RNA of cells was extracted by one-step method of Trizol-isoamyl alcohol, and synthesized to cDNA by two-step reverse transcription. Using the synthesized cDNA as a template, the PCR reaction was performed by SYBR Premix Ex Taq II. The reaction conditions were: predenaturation at 95 °C for 5 min; 40 cycles of denaturation at 94 °C for 15 s, annealing at 55 °C for 30 s, and elongation at 70 °C for 30 s; final elongation at 70 °C for 10 min. The primers sequences were: CD59 sense: 5′-CAAGGAGGGTCTGTCCTGTT-3′, anti-sense: 5′-GACCTGAATGGCAGAAGACA-3′; Bcl-2 sense: 5′-GAGACAGCCAGGAGAAATCAA-3′, anti-sense: 5′-ATGTGTGTGGAGAGCGTCAA-3′; Bax sense: 5′-CTCAGCCCATCTTCTTCCAG-3′, anti-sense: 5′-AGCGACTGATGTCCCTGTCT-3′; actin sense: 5′-CGTGGAAGGACTCATGACCA-3′, anti-sense: 5′-TCCAGGGGTCTTACTCCTTG-3′. The relative quantification was identified by the 2^−∆∆Ct^ method after standardization to the actin level.

### Enzyme linked immunosorbent assay (ELISA)

According to the manufacturer’s instructions of the Human IL-2 ELISA Kit (ab174444, Abcam, USA), the IL-2 content in the cell culture medium was examined.

### Establishment of a nude mouse model of T-ALL

Thirty-two 4-week-old BALB/c-nu female mice, weighting 18–22 g, were purchased from the Slyke Animals Corporation of the Chinese Academy of Sciences (Shanghai, China), and randomly divided into four groups: Blank (NC), Jurkat, wild type (WT) and mutant1 (M1) groups. The mice of last three group were injected intraperitoneally with 100 mg/kg cyclophosphamide (CTX), while mice of the NC group were injected intraperitoneally with equal volume of physiological saline, for 2 consecutive days. Subsequently, the mice of Jurkat, WT and M1 groups respectively received tail vein injections of 1 × 10^7^/0.2 ml normal Jurkat cells, WT Jurkat cells and M1 Jurkat cells, and the mice in the NC group were tail vein injected with the same amount of physiological saline, continuously for 2 days. Before the cells were injected and within 4 weeks after the cells were injected, the experimenters observed and measured the state of the mice, and recorded each day.

### Mouse peripheral white blood cell count

Before the cells were injected and at the 1, 2, 3 and 4 weeks after the cells were injected, blood was collected from tail veins of each group for peripheral blood leukocyte counts. The specific procedures were as follows: The tail was broken, and 20 μl of peripheral blood was pipetted accurately into the test tube. Further add 380 μl white cell diluent, and shake gently. 10 μl of cell suspension was taken into a counting plate, and counted after standing for 2–3 min. Under the microscope, the shape of white blood cells was round, the pulp was translucent, and the nucleus was purple-black.

### Detection of IL-2 level in serum by ELISA

The mice were anaesthetized and dissected to expose the heart. The blood was taken from the apex with a 5 ml syringe, and slowly placed in a clean centrifuge tube to prevent hemolysis. Store at room temperature for 4 h, and centrifuge at 150×*g* for 15 min. The light yellow supernatant was the desired mouse serum. The determination of IL-2 in serum was same as that in culture medium.

### Apoptosis detection in bone marrow and peripheral blood of mice

The pretreatment step of bone marrow: the femur of the mouse’s lower limb was taken and cut from both ends. PBS solution was extracted with 1 ml sterile syringe to wash the medullary cavity repeatedly. Rinsed bone marrow fluid was added 3 times the volume of red blood cell lysate and gently vortex or invert. Then incubate on ice for 15 min and shake it from time to time. Whereafter, the mixture was filtered through a 200-mesh screen, and the filtered cell suspension was centrifuged at 450×*g* for 10 min. Carefully and thoroughly remove the supernatant, add twice volume of red blood cell lysate, and centrifuge under the same conditions. Finally, the cells were resuspend by 100 μl of Binding buffer, and apoptosis were detected as described above. The blood taken from tail vein must be processed within 6 h. the treatment and detection methods were the same as for bone marrow.

### CD59 knockdown Jurkat cells

The shRNA of CD59 was ligated to the pLKO.1-puro-CMV-TurboGFP (VT8114, YouBio, China) lentiviral vector, and the recombinant lentivirus were collected and concentrated. Then, infect Jurkat cells to knockdown the expression of CD59 (KD). The shRNA sequence was as follows: 5′-CCGGCCGTCAATTGTTCATCTGATTCTCGAGAATCAGATGAACAATTGACGGTTTTTG-3′.

### Statistical analysis

All experimental data were statistically analyzed using SPSS 19.0 software. The data were expressed as mean ± standard deviation (X ± SD). The comparison of multiple groups was performed using one-way analysis of variance, while that between the two groups was performed using t-test of independent samples. P < 0.05 was considered statistically significant.

## Results

### CD59 is highly expressed in bone marrow of T-ALL patients

**Fig. 1:**
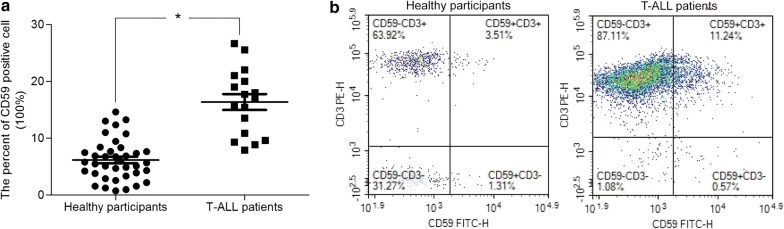
CD59 is highly expressed in bone marrow of patients with T-ALL. **a** Scatter plot of CD59 positive percent of bone marrow samples in 17 T-ALL patients and 38 healthy participants. **b** An example of flow diagram for CD3 and CD59 analysis. All experiments were performed three times. *P < 0.05

First of all, we performed the CD59 positive analysis in bone marrow samples collected from 17 T-ALL patients and 38 healthy individuals. As shown in Fig. 1a, the proportion of CD59^+^ T lymphocytes in bone marrow of T-ALL patients was significantly higher than that in healthy individuals (16.37 ± 1.397% vs 6.168 ± 0.5818%, *P* < 0.0001). And statistical analysis (Tables 3 and 4) showed that the proportion of CD59^+^ T lymphocytes in T-ALL patients was not related to age (*P* = 0.3607, R^2^ = 0.05594), gender (*P* = 0.2932) and disease stage (*P* = 0.0816). The proportion in healthy individuals was also independent of age (*P* = 0.2466, R^2^ = 0.03711) and gender (*P* = 0.2597).

**Table 3:** Correlation between CD59^+^ T lymphocyte ratio and age

Participants	Age							P	R^2^
	≤ 10	~ 20	~ 30	~ 40	~ 50	~ 60	> 60		
T-ALL (num = 17)	1	4	3	3	2	3	1	0.3607	0.05594
Healthy (num = 38)	0	9	7	6	5	3	8	0.2466	0.03711

**Table 4:** Clinical samples information

Clinicopathological parameters	T-ALL (num = 17)	P	Healthy (num = 38)	P
Stage		0.0816		
L1	11		0	
L2	6		0	
L3	1		0	
Gender		0.2932		0.2597
Male	13		23	
Female	4		15	

### The expression of wild-type CD59 is beneficial to the survival of Jurkat cells

Further, we aimed to study if the change in the levles of CD59 was able to affect the survival of T-ALL cell lines. Briefly, four Jurkat cell lines were designed and constructed using directed mutation technology, namely, stable expression of wild-type human CD59 (WT), stable expression of W40 mutation human CD59 (M1), stable expression of K41 mutation human CD59 (M2) and infected with a lentivirus empty vector as a negative control (NC). Fluorescence microscopy, flow cytometry analysis, real-time quantitative PCR and western blot results (Additional file 1: Figure S1) showed that we have successfully constructed Jurkat cells stably expressing wild-type or mutant human CD59.

**Fig. 2:**
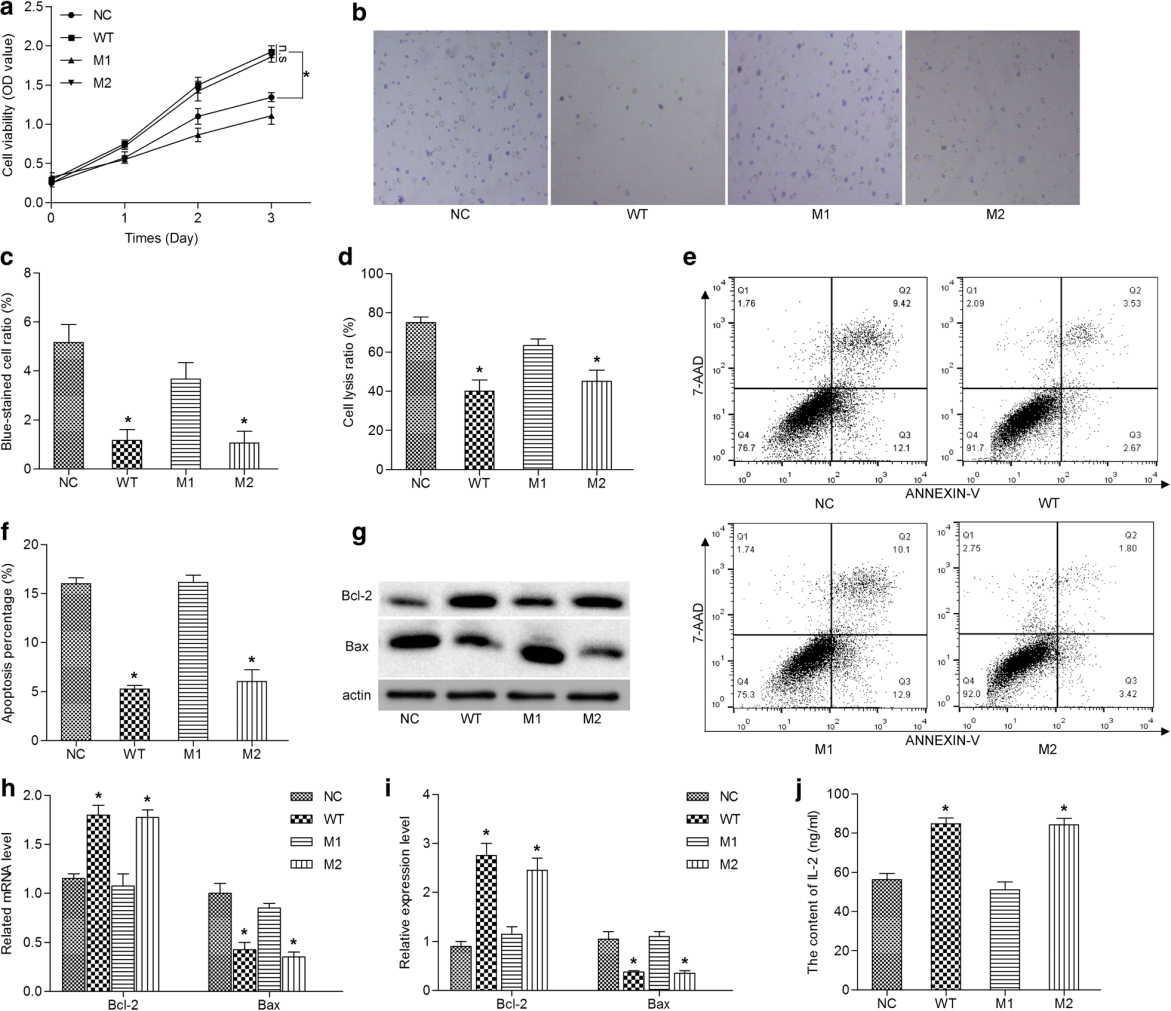
The expression of wild-type CD59 is beneficial to the survival of Jurkat cells. **a** The cell vitality detected by CCK8 assay. **b** The dead cells detected by trypan blue staining. **c** Quantitative analysis of blue-stained cells. **d** Lytic cells detected by Dye release assay. **e** The apoptosis detected by flow cytometry. **f** Quantitative analysis of apoptosis. **g** The protein expression of Bax and Bcl-2 detected by western blot. **h** Quantitative analysis of mRNA expression of Bax and Bcl-2. **i** Quantitative analysis of protein expression of Bax and Bcl-2. **j** The content of IL-2 in culture medium detected by ELISA. All experiments were performed three times. *P < 0.05. *n.s* no significant

CCK8 assays showed that compared with NC group, the OD values of WT group and M2 group were markedly increased (*P *< 0.05) on the 3 day, and there was no significant difference between M1 group and NC group (Fig. 2a). Interestingly, the OD values of M1 group were significantly lower compared to WT and M2 groups (*P* < 0.05, Fig. 2a). Similarly, blue trypan exclusion assay showed a significant decrease in the proportion of dead cells in WT and M2 groups compared with the NC group (*P *< 0.05), and there was no significant difference in M1 group (*P* > 0.05, Fig. 2b, c). Compared with the WT and M2 groups, the cell death rate was significantly higher in the M1 group (*P *< 0.05, Fig. 2b, c). These results revealed that the expression of wild-type and M2 mutant CD59 favored survival of Jurkat cells; whereas the expression of M1 mutant CD59 had no significant effect on cell survival. These conditions led us to speculate that the W40 was a key site for the function of CD59, while K41 was not.

It was well known that CD59 was closely related to the complement-mediated cytolysis. The results of dye release experiments also reinforced this argument (Fig. 2d). At the same dilution of complement, cells which in WT and M2 groups with lower dye release rate were insensitive to complement-mediated cytolysis than NC group (*P *< 0.05, Fig. 2d). The dye release rate of the M1 group was not significantly different from that of the NC group, but significantly higher than that of the WT and M2 groups (*P* < 0.05, Fig. 2d). Further, we also found that the effect of CD59 on Jurkat cells survival involved the regulation of apoptosis. As shown in Fig. 2e, f, the apoptosis rates of WT and M2 groups were decreased compared to NC group (*P *< 0.05), meantime, the apoptosis rate of M1 group was not strongly diverse from that of NC group, but increased than WT and M2 groups (*P *< 0.05). These results indicated that CD59 not only involved in complement-mediated cytolysis, but also participated in apoptosis; during this process, the W40 remained a critical site.

Bcl-2 and Bax were important apoptosis-related proteins, and the main role of IL-2 was to promote lymphocyte growth, proliferation, and differentiation. In previous experiments, we had found that CD59 was involved in the proliferation, growth and apoptosis of Jurkat cells. Therefore, we further examined the effects of CD59 on Bcl-2 and Bax expression, IL-2 production and secretion. As shown in Fig. 2g–i, these changes in molecular levels were consistent with changes in cell biology. Compared with the NC group, the mRNA and protein levels of Bcl-2 were significantly up-regulated in the WT and M2 groups (*P *< 0.05), and that of Bax were downregulated (*P *< 0.05), while the expression of Bcl-2 and Bax in the M1 group did not change meaningfully. However, the expression of Bcl-2 was decreased, and the expression of Bax was enhanced in M1 group compared with WT and M2 groups (*P *< 0.05). In addition, the IL-2 content in the cell culture medium was detected by ELISA. Compared with NC group, IL-2 levels were elevated in the WT and M2 groups, and these was no significant difference in M1 group (*P *< 0.05, Fig. 2j). And the IL-2 levels of M1 group were significantly lower than that of the WT and M2 groups (*P *< 0.05, Fig. 2j). In conclusion, we found that the expression of CD59 in Jurkat cells not only inhibited complement-mediated cytolysis, but also prevented the apoptosis and promoted IL-2 secretion, thereby helping cells to survive better. The W40 site played an indispensable role.

### CD59 inhibits apoptosis in mouse model

**Fig. 3:**
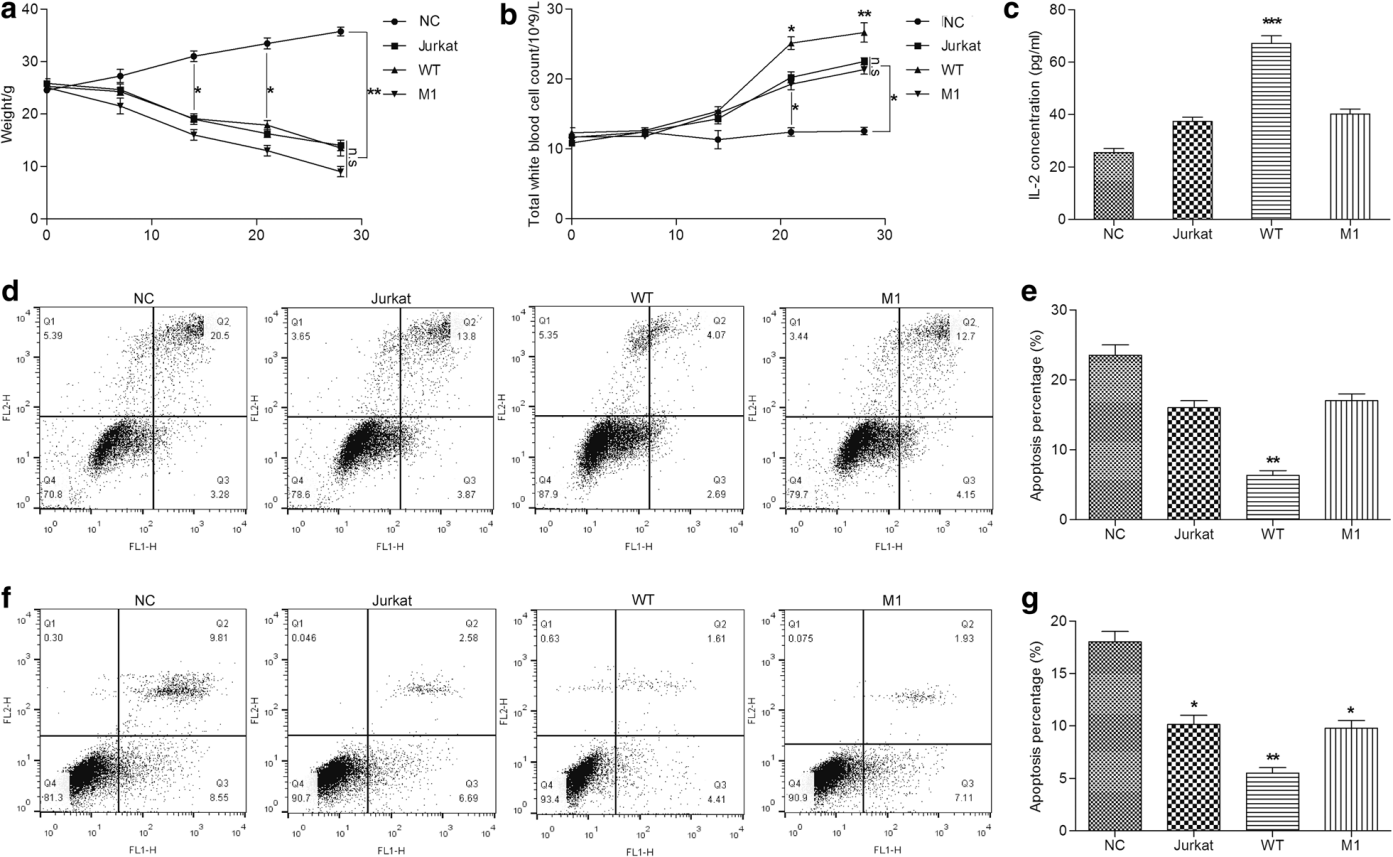
CD59 inhibits apoptosis in mouse model. Body weight (**a**) and white blood cell count in peripheral blood (**b**) of mouse model in 0, 1, 2, 3, 4 weeks. **c** The IL-2 levels in peripheral blood detected by ELISA. The apoptosis in bone marrow (**d**) and peripheral blood (**f**) detected by flow cytometry. Quantitative analysis of apoptosis in bone marrow (**e**) and peripheral blood (**g**). All experiments were performed three times. *P < 0.05, **P < 0.001, ***P < 0.0001. *n.s* no significant

In addition, we aimed to confirm the effect of CD59 in T-ALL mouse models. Before the experiment, all nude mice were healthy, lively and energetic. During the tail vein injection, two mice in Jurkat group, three mice in WT group, and one mice in M1 group died. Except the NC group, mice in each group showed varying degrees of dietary loss, decreased body weight, growth retardation, fur shrinkage, lethargy and bow back, and so on. The results of body weight weighting were shown in Fig. 3a, mice in NC group were injected with physiological saline, fed and water normally, and their body gradually increased with time. After the third week, the body weight stabilized. In the initial phase of modeling (first week), the body weight of Jurkat and M1 groups did not decrease significantly, and decreased significantly after 1 week. There was no significant difference in body weight between the two groups. The weight loss of the WT group was essentially the same as that of the Jurkat group, but the average body weight was smaller. The results of white blood cell counts in peripheral blood were shown in the Fig. 3b. The number of white blood cells in the Jurkat, WT and M1 groups did not change significantly during the initial period of modeling (the first 2 weeks), but rapidly increased after 2 weeks. There was no significant difference in peripheral blood leukocyte counts between Jurkat and M1 group. And the number of peripheral blood leukocytes in WT group was significantly higher than that in Jurkat and M1 groups after 2 weeks of modeling. IL-2 levels in peripheral blood were shown in Fig. 3c. Serum IL-2 levels were no significantly difference in Jurkak and M1 group compared with NC group, while the serum IL-2 levels of WT group were significantly elevated (*P *< 0.0001).

Bone marrow was the main hematopoietic and immune organ, containing hematopoietic stem cells and a variety of other stem cells that can differentiate into various cells, such as red blood cells, granulocytes, monocytes, and macrophages. We used flow cytometry to detect the apoptosis in bone marrow. The apoptosis rate in bone marrow of WT group was significantly lower than that of the other three groups (*P *< 0.05, Fig. 3d, e). The percentage of apoptosis in peripheral blood was similar to that in bone marrow, which was significantly lower in Jurkat and M1 group than that of NC group (*P *< 0.05, Fig. 3f, g), and the apoptosis rate in WT group was lower in this basis (*P *< 0.001, Fig. 3f, g). These data suggested that high expression of CD59 inhibited apoptosis of bone marrow and peripheral blood, and promoted IL-2 secretion in mouse model. During this process, the W40 site also played an indispensable role.

### The activation of AKT, STAT5 and Notch1 signaling pathway are involved in the regulation of apoptosis by CD59 in Jurkat cells

In the previous part of this study, we found that CD59 was highly expressed in bone marrow cells of T-ALL patients, and that overexpression of CD59 in Jurkat cells could inhibit apoptosis in cell experiments and mouse models. In this process, W40 site played an important role. Further, we continued to explore the molecular mechanisms of the impact of CD59 expression and W40 mutant on apoptosis, and to search for related downstream signaling pathways.

**Fig. 4:**
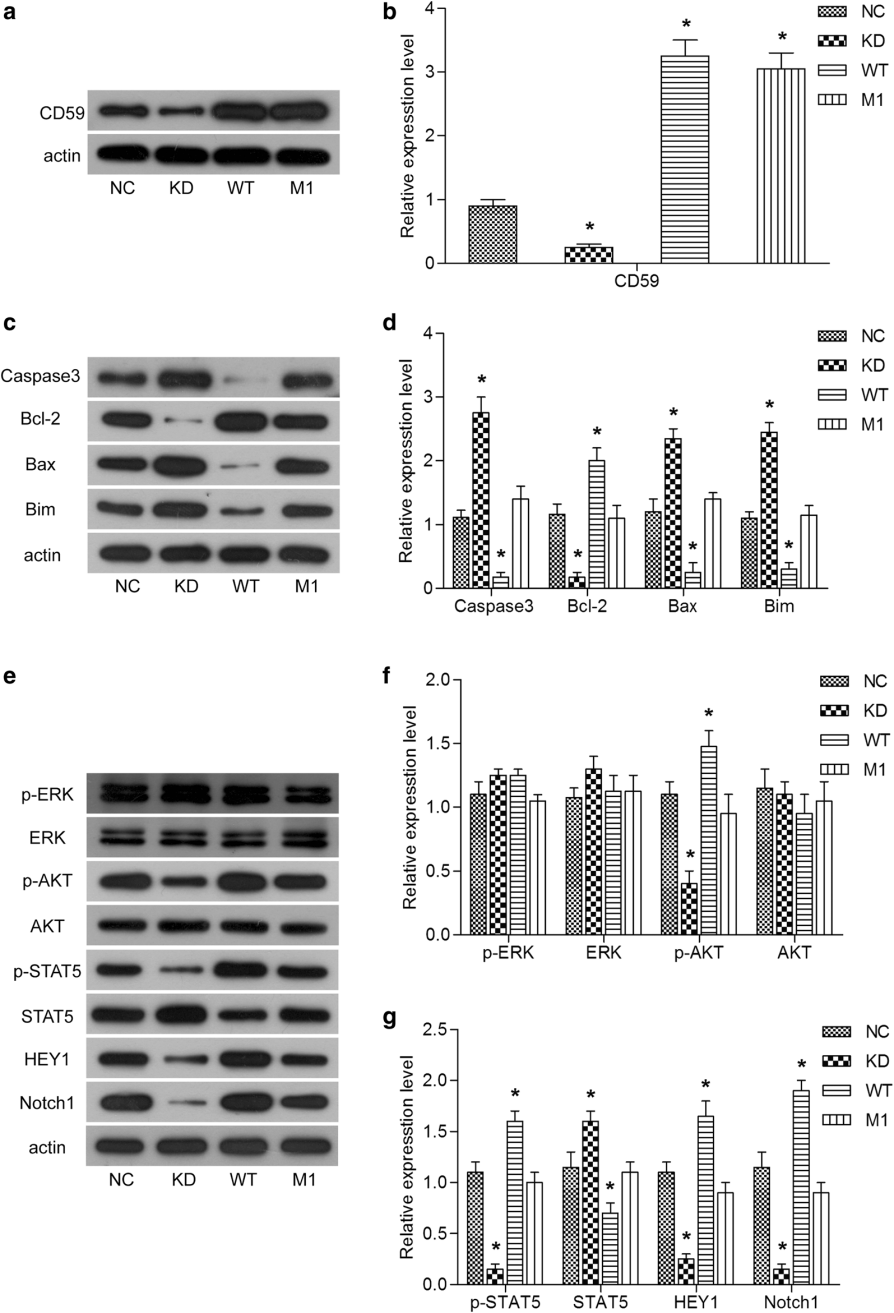
The activation of AKT, STAT5 and Notch1 signaling pathway are involve in the regulation of apoptosis by CD59 in Jurkat cells. The expression of CD59 (**a**), apoptosis-related proteins (**c**) and key proteins in signaling pathways (**e**) detected by western blot. Quantitative analysis of the protein expression of CD59 (**b**), apoptosis-related proteins (**d**) and key proteins in signaling pathways (**f**, **g**). All experiments were performed three times. *P < 0.05

First, the results of western blot showed the expression of CD59 in each group (Fig. 4a, b). The expression of apoptosis-related proteins in each group was examined, and the results were shown in the Fig. 4c, d. When CD59 was down-regulated, the expression of pro-apoptotic proteins, Caspase 3, Bax, Bim, was up-regulated, and the expression of anti-apoptotic protein Bcl-2 were down-regulated. In contrast, the levels of Caspase 3, Bax and Bim were decreased, while the levels of Bcl-2 were increased in WT group. And there was no significant difference in the expression of apoptosis-related proteins between the M1 group and the NC group. These results indicated that the expression of CD59 and the presence of W40 in Jurkat cells were closely associated with the expression of apoptosis-related proteins.

Through the above experiments, we had determined that CD59 could regulate apoptosis by regulating the expression of apoptosis-related proteins. To explore its molecular mechanisms, we investigated several key proteins involved in cell proliferation, differentiation, and apoptosis, including ERK, AKT, STAT5, HEY1, and Notch1. As shown in Fig. 4e, g, there was no significant change in the expression of p-ERK and ERK in each group. There was also no significant difference in the expression of AKT. However, the level of p-AKT was decreased in CD59 knock-down cells, and increased in CD59-overexpressing cells compared with NC group. The level of p-AKT protein in M1 group were found no significant change compared with NC group. These results suggested that the expression of CD59 and the presence of W40 site in Jurkat cells were related to the phosphorylation of AKT. Consistent with the trend of p-AKT, p-STAT5 was also significantly down-regulated by CD59 knockdown and up-regulated by CD59 overexpression; in cells with W40 mutation in CD59, there was no significant change of p-STAT5 protein levels compared with NC group. It was demonstrated that the expression of CD59 and the presence of W40 in Jurkat cells were associated with the phosphorylation of STAT5. In addition, when CD59 was down-regulated, the expression of Notch1 and its target gene HEY1 was down-regulated. When CD59 was highly expressed, the expression of Notch1 and HEY1 was up-regulated. Compared with WT group, the expression level of Notch1 and HEY1 was down-regulated in M1 group. It was demonstrated that in Jurkat cells, the expression of CD59 and the presence of W40 site were implicated in the expression of Notch1 and its downstream target proteins. These results revealed that the activation of AKT, STAT5 and Notch1 signaling pathway in Jurkat cells, may be involved in the regulation of apoptosis by CD59; and mutation at the W40 site affected the interaction of CD59 with these signaling pathways.

### CD59 regulates apoptosis through AKT/Notch1 signaling pathway

**Fig. 5:**
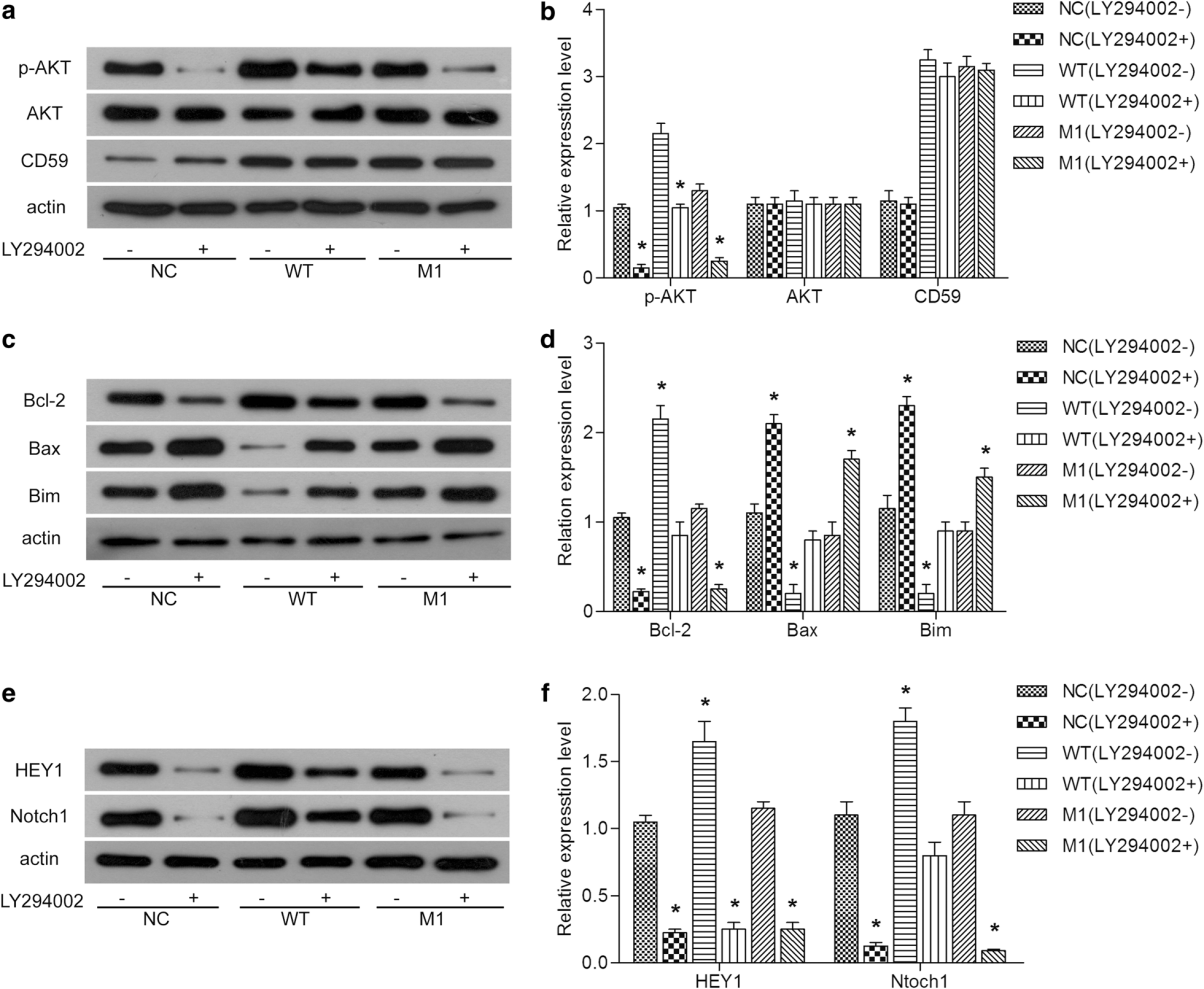
CD59 regulates apoptosis through AKT/Notch1 signaling pathway. The expression of CD59, AKT and p-AKT (**a**), apoptosis-related proteins (**c**) and key proteins in Notch1 signaling pathways (**e**) detected by western blot. Quantitative analysis of the protein expression of CD59, AKT and p-AKT (**b**), apoptosis-related proteins (**d**) and key proteins in Notch1 signaling pathways (**f**). All experiments were performed three times. *P < 0.05

We performed a compensation experiment using the AKT specific inhibitor LY294002 (HY-10108, MedChemExpress, USA) which completely inhibited the phosphorylation of AKT in HepG2 cells. As could be seen from the Fig. 5a, b, the addition of LY294002 (5 μM) effectively inhibited the phospholylation of AKT. And LY294002 up-regulated the expression of Bax and Bim, and inhibited the expression of Bcl-2 (Fig. 5c, d). Moreover, LY294002 efficiently inhibited the expression of Notch1 and HEY1 in each group (Fig. 5e, f). This indicated that CD59 regulated apoptosis through AKT/Notch1 signaling pathway in Jurkat cells.

## Discussion

CD59 is a GPI-anchored glycoprotein that inhibites MAC formation in cell surface [15]. At the same time, CD59 acts as a second signal stimulator to induce T cell activation and is participated in the regulation of the immune respone [16, 21, 22]. The reduction of CD59 expression on T cells surface markedly enhances the activation and proliferation of CD4^+^ and CD8^+^ T cells [23]. Binding of CD59 to monoclonal antibodies leads to phosphorylation of TCRγ/ZAP70 subunits and secretion of IL-2, further promoting the proliferation and activation of T-cells [24, 25]. However, in this study, we found that the proportion of T lymphocytes expressing CD59 in bone marrow of T-ALL patients was significantly higher than that of heathy individuals. Of course, we must also note that the 17 T-ALL patients in this study is too low and need to be further expanded.

Previous studies had shown that CD59 was highly expressed in a variety of solid tumors and cells, acting as a complement regulatory protein that prevented MAC formation by binding to C8 or C9, thereby protecting tumor cells from complement-mediated cytolysis and evading immune detection. The dye release experiments and trypan blue staining experiments performed in this study confirmed that overexpression of CD59 in Jurkat cells reduced the sensitivity to complement-mediated cytolysis and cell death rate. There was no significant change in complement sensitivity and cell death rate in Jurkat cells with CD59 K41 mutation. However, Jurkat cells with CD59 W40 mutation had reduced complement sensitivity, and a significant increase in cell death. These results indicated that the W40 was a critical functional site during the anti-complement effect of the CD59.

In this study, we found that overexpression of CD59 in Jurkat cells promoted cell proliferation and reduced apoptosis. When CD59 was knocked down, the expression of Caspase 3, Bax and Bim were up-regulated, and the expression of Bcl-2 was down-regulated. The expression of Caspase 3, Bax and Bim were down-regulated, and the expression of Bcl-2 was up-regulated, when CD59 was overexpressed in Jurkat cells. More interestingly, the W40 site still played a key role in this process. The correlation between CD59 and tumor cell apoptosis had been studied in Hela, GLC-P, MCF-7 and HT-29 cells [10, 11, 26–28] These studies suggested that overexpression of CD59 in these tumor cells promoted the cell proliferation and inhibited apoptosis, while silencing of CD59 inhibited cell proliferation and promoted apoptosis. The silencing of CD59 in HT-29 cells promoted cell sensitivity to the chemotherapeutic drugs 5-fluorouracil and oxaliplation by promoting apoptosis and blocking G0/G1 phases, which provided a guidance for combination chemotherapy of colorectal cancer [27]. CD59 might be applied in the clinical treatment of T-ALL and other tumors in the near future. The study of aberrant activation of signal transduction through cell–cell interactions was an emerging topic in leukemia cell biology. Growth factors secreted by cells protected leukemia cell populations. Several researches had shown that the proliferation and survival of T-ALL cells depended on cytokines, such as IL-2, IL-4, IL-8, IL-9, and IL-15 [29–32] In this study, the concentration of IL-2 in CD59-overexpressing Jurkat cell cultures was increased by ELISA compared to normal Jurkat cells. Compared with CD59-overexpressing Jurkat cells, the W40 mutation inhibited the secretion of IL-2, whereas the K41 mutation did not affect IL-2 secretion. These data suggested that CD59 exerted multiple functions in Jurkat cells, including complement regulatory already deeply studied, as well as apoptosis regulatory shallowly understood, and the effect on IL-2 secretion, meantime W40 was essential in these processes.

The establishment of leukemia animal models contributed to the study of human lymphocytic leukemia. In this research, an expression plasmid of wild-type CD59 or W40-mutant CD59 was constructed, and injected into Juakat cells by lentivirus. The infected Jurkat cells were infected into the nude mice by tail vein to construct a T-ALL model. The constructed model showed decreased appetite, skin folds, roach back, and decreased body mass. The number of white blood cells in peripheral blood increased significantly, and the secretion of IL-2 was increased in constructed model. The high expression of CD59 significantly inhibited apoptosis of bone marrow and peripheral blood. Although CD59 was overexpressed, there was no significant difference between the mouse model of W40-mutant and Jurkat groups.

Targeting signal pathways that were mutated or over-activated during the onset and progression of T-ALL became a new therapeutic strategy, currently included Ras/Raf/MEK/ERK, PI3K/AKT/mTOR, IL-7/JAK/STAT5, and Notch1 signaling pathways [33–39] Activation of ERK was almost present in all T-ALL samples, and activation of MEK/ERK was an independent negative prognostic indicator for T-ALL patients [33, 37]. In this study, knockdown or overexpression of CD59 in Jurkat cells did not cause significantly changes in the expression of ERK and p-ERK, suggesting that ERK signaling may not be involved in the regulation of Jurkat cells behavior by CD59. The PI3K/AKT/mTOR signaling pathway was involved in extensive intracellular phosphorylation, and was found to be widely activated in a variety of tumor types. The runaway of the PI3K/AKT/mTOR signaling pathway in a series of leukemias, including T-ALL, had led to the development of cancer [34–36] A study using zebrafish as a living model had demonstrated that the AKT pathway played a key role in the expansion of the leukemia cell pool and eventually led to hematologic recurrence [40]. In this study, we found that knockdown of CD59 in Jurkat cells inhibited phosphorylation of AKT, whereas overexpression of CD59 promoted phosphorylation of AKT. And mutation of W40 also affected phosphorylation of AKT. And the IL-7 receptor signaling pathway was tightly regulated in normal T cells because it is necessary for progenitor T- cell proliferation and survival [38]. The binding of the heterodimeric IL-7 receptor to IL-7 induced the phosphorylation of JAK1 and JAK3 [39]. Then, dimerized STAT5 migrated to the nucleus and regulated the expression of the target gene [39]. The anti-apoptotic effect of this pathway was mainly through inhibiting the expression of Bcl-2 [39]. Activation of JAK and STAT5 was a common feature of immature leukemia [41, 42]. Our study found that knockdown of CD59 in Jurkat cells inhibited STAT5 activation, whereas overexpression of CD59 promoted STAT5 activation. And mutation of W40 in CD59 inhibited phosphorylation of STAT5. At last, the most common mutation in T-ALL was the Notch1 gene mutation and the deletion of chromosome 9/CDKN2A [43–45] Activation of Notch1 and inactivation of Notch1 negative regulator FBXW7 were found in 60% of T-ALL cases [46]. Our study found that knockdown of CD59 in Jurkat cells down-regulated the expression of Notch1 and its target gene HEY1, whereas the expression of Notch1 and HEY1 was up-regulated when CD59 was overexpressed. The mutation of W40 inhibited the expression of Notch1 and HEY1. These data revealed that the expression of CD59 could affect the activation of AKT, STAT5, Notch1 and HEY1.

Recent studies had found a correlation between Notch1 and PI3K/AKT/mTOR1 signaling pathways in T-ALL cells [47–52] Notch1 could increase the expression of growth factor receptors (IGF1R and IL-7R), and decrease the p53 and PTEN levels in T-ALL cells, which was beneficial to the activation of AKT [47–52] In this study, we found that the addition of the AKT specific inhibitor LY294002 effectively inhibited the activation of AKT, promoted the apoptosis, and inhibited the expression of Notch1 and HEY1, which implied that the link between Notch1 and AKT signaling pathways was complex. After the addition of LY294002, the expression of Bcl-2 was down-regulated, whereas the expression of Bax and Bim was up-regulated. And the expression of Notch1 and HEY1 was decreased. These results indicated that AKT signaling may directly or indirectly affect the regulation of apoptosis by CD59 through Notch1 signaling in Jurkat cells.

## Conclusion

In conclusion, CD59 inhibited apoptosis of T-ALL by regulating AKT/Notch1 signaling pathway, providing a new perspective for the treatment of T-ALL.

## Additional file

**Additional file 1: Figure S1.** Wild and mutant CD59-expressing Jurkat cells were successfully constructed. (**A**) The green fluorescence detected by fluorescence microscope X100. (**B**) The transfection efficience detected by flow cytometry. The protein (**C**) and mRNA (**D**) levels of CD59 detected by qRT-PCR in NC, WT, M1 and M2 group. (**E**) Quantitative analysis of the protein expression of CD59. All experiments were performed three times. **P < 0.001, ***P < 0.0001.
