# Supplementary figures and images for: Overexpression of CD59 inhibits apoptosis of T-acute lymphoblastic leukemia via AKT/Notch1 signaling pathway
Source: Cancer Cell Int. 2019 Jan 8;19:9. doi: 10.1186/s12935-018-0714-9 (PMC6325688; doi:10.1186/s12935-018-0714-9)

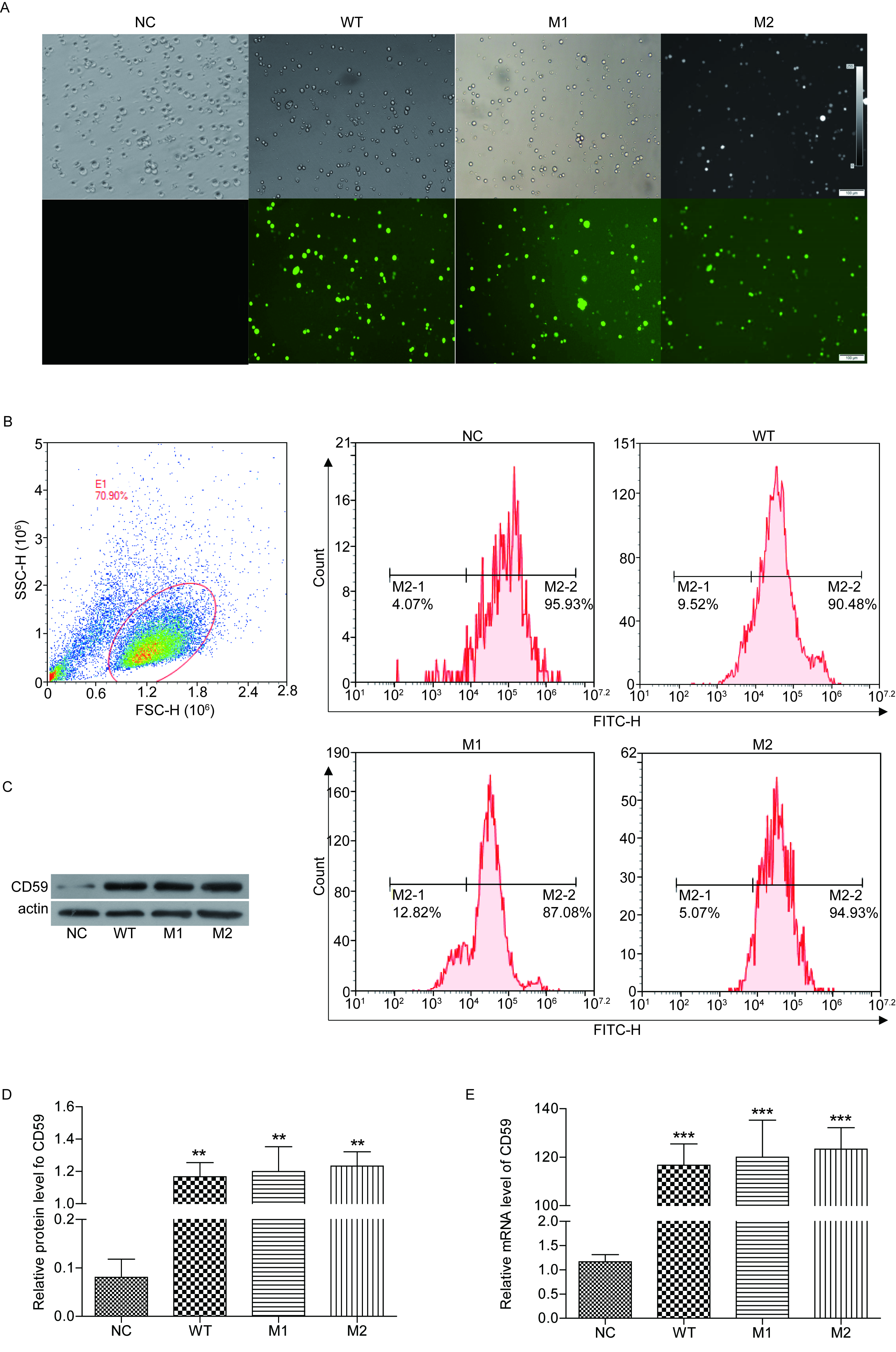

Supplement: Supplementary file 1 — Additional file 1: Figure S1. Wild and mutant CD59-expressing Jurkat cells were successfully constructed. (A) The green fluorescence detected by fluorescence microscope X100. (B) The transfection efficience detected by flow cytometry. The protein (C) and mRNA (D) levels of CD59 detected by qRT-PCR in NC, WT, M1 and M2 group. (E) Quantitative analysis of the protein expression of CD59. All experiments were performed three times. **P < 0.001, ***P < 0.0001. [file 12935_2018_714_MOESM1_ESM.tif]
